# Treatment with gelsolin reduces brain inflammation and apoptotic signaling in mice following thermal injury

**DOI:** 10.1186/1742-2094-8-118

**Published:** 2011-09-21

**Authors:** Qing-Hong Zhang, Qi Chen, Jia-Rui Kang, Chen Liu, Ning Dong, Xiao-Mei Zhu, Zhi-Yong Sheng, Yong-Ming Yao

**Affiliations:** 1 Department of Microbiology and Immunology, Burns Institute, First Hospital Affiliated to the Chinese PLA General Hospital, Beijing 100048, PR China; 2 Department of Pathology, First Hospital Affiliated to the Chinese PLA General Hospital, Beijing 100048, PR China; 3 Undergraduate Medical School, 4th Military Medical University, Xi'an, Shaanxi, 710032, PR China; 4 State key laboratory of kidney disease, the Chinese PLA General Hospital, Beijing 100853, PR China

**Keywords:** Burns, Gelsolin, Septic encephalopathy, Neuroinflammation, Caspase-3, Apoptosis

## Abstract

**Background:**

Burn survivors develop long-term cognitive impairment with increased inflammation and apoptosis in the brain. Gelsolin, an actin-binding protein with capping and severing activities, plays a crucial role in the septic response. We investigated if gelsolin infusion could attenuate neural damage in burned mice.

**Methods:**

Mice with 15% total body surface area burns were injected intravenously with bovine serum albumin as placebo (2 mg/kg), or with low (2 mg/kg) or high doses (20 mg/kg) of gelsolin. Samples were harvested at 8, 24, 48 and 72 hours postburn. The immune function of splenic T cells was analyzed. Cerebral pathology was examined by hematoxylin/eosin staining, while activated glial cells and infiltrating leukocytes were detected by immunohistochemistry. Cerebral cytokine mRNAs were further assessed by quantitative real-time PCR, while apoptosis was evaluated by caspase-3. Neural damage was determined using enzyme-linked immunosorbent assay of neuron-specific enolase (NSE) and soluble protein-100 (S-100). Finally, cerebral phospho-ERK expression was measured by western blot.

**Results:**

Gelsolin significantly improved the outcomes of mice following major burns in a dose-dependent manner. The survival rate was improved by high dose gelsolin treatment compared with the placebo group (56.67% vs. 30%). Although there was no significant improvement in outcome in mice receiving low dose gelsolin (30%), survival time was prolonged against the placebo control (43.1 ± 4.5 h vs. 35.5 ± 5.0 h; P < 0.05). Burn-induced T cell suppression was greatly alleviated by high dose gelsolin treatment. Concurrently, cerebral abnormalities were greatly ameliorated as shown by reduced NSE and S-100 content of brain, decreased cytokine mRNA expressions, suppressed microglial activation, and enhanced infiltration of CD11b+ and CD45+ cells into the brain. Furthermore, the elevated caspase-3 activity seen following burn injury was remarkably reduced by high dose gelsolin treatment along with down-regulation of phospho-ERK expression.

**Conclusion:**

Exogenous gelsolin infusion improves survival of mice following major burn injury by partially attenuating inflammation and apoptosis in brain, and by enhancing peripheral T lymphocyte function as well. These data suggest a novel and effective strategy to combat excessive neuroinflammation and to preserve cognition in the setting of major burns.

## Background

Brain is one of the remote organs subjected to injurious effects of severe burns [1]. Survivors suffering from extensive burn injury present long-term cognitive impairment, including depression, anxiety, post-traumatic stress disorder [2,3], and alteration in painful sensation as well as sensory sensitivity in later life [4]. In animal studies, magnetic resonance imaging has identified marked changes in the brain up to 3 days postburn (pb), most notably swelling and lesions [5], changes in cerebral blood flow [6], dysregulation of glucose metabolism [7], and disruption of the blood-brain barrier (BBB) [8,9].

Neuroinflammation is a frequent consequence of sepsis and septic shock [10]. Approximately 93% of burn patients show clinical signs of a systemic inflammatory response syndrome before succumbing to their injuries [11], and this syndrome can deteriorate and develop into severe sepsis [12]. After burn injury, there is a dramatic increase in proinflammatory cytokines in brain as early as 3 hours (h) [13,14] and a compromised BBB leading to a large infiltration of macrophages [9]. Beneficial as well as deleterious effects have been ascribed to immune cells that infiltrate the nervous system after neural injury [15-19]. Despite the correlation between cerebral complications in severe burn victims and mortality, burn-induced neuroinflammation continues to be an underestimated entity in critically ill burn patients [10].

Gelsolin was first described as a ~90 kDa cytoplasm actin-binding protein with capping and severing activities [20]. Further studies have confirmed a secreted gelsolin isoform in blood plasma [21]. Recent reports have documented that it also participates in the regulation of the systemic immune response. Extracellular gelsolin is involved in host immune recognition of bacterial wall molecules during cell division or attack by immune components, while cytoplasmic gelsolin is necessary for macrophage motility in culture, and its absence is likely to impair recruitment of macrophages to a site of crush injury of sciatic nerve [22]. In fact, overexpression of gelsolin could alter actin dynamics in Jurkat T cells, correlating with inhibition of activation-dependent signaling pathways [23]. Moreover, cytoplasmic gelsolin depletion is observed in diverse states of inflammation that are associated with tissue injury and actin release, including hemorrhagic shock [24], early sepsis, trauma, and rheumatoid arthritis [25]. In addition, its deficiency has been found to correlate with septic mortality [26] and prognosis [27], suggesting that gelsolin might play a crucial protective role in the course of sepsis.

Accordingly, gelsolin replacement might be considered as a potential therapy for the lethal condition of sepsis [28]. It could solubilize circulating actin aggregates and shift expressed cytokines toward an anti-inflammatory profile [28], resulting in a significant reduction of mortality in endotoxemic mice. Since gelsolin has been shown to significantly blunt neutrophil recruitment to lungs [29] and to markedly attenuate vascular permeability in burn injury in rats [30], we hypothesized that, in severe burn injury of mice, a single dose of gelsolin might attenuate neuroinflammation, which might ultimately protect the brain from injurious effects following the acute insult.

## Methods

### Animal model of burn injury

Male Balb/c mice (20-25 g, 8-9 weeks old, obtained from the Laboratory Animal Institute, Beijing, China) were anesthetized, and the dorsal and lateral surfaces of the mice were shaved. Mice were secured in a protective template on their backs with an opening corresponding to 15% of the total body surface area (TBSA), and the exposed skin was immersed in 95°C water for 8 seconds (s). This procedure has been shown to produce a 15% TBSA full-thickness scald injury. Sham-injured mice were subjected to all of the procedures except that the temperature of the bath was the same as room temperature. Immediately following injury, the mice were dried and allowed to recover under a heating lamp. Both sham- and burn-injured mice received 1.0 ml of fluid for resuscitation intraperitoneally (i.p.) (Ringer's solution). Animals were then housed in individual cages in a temperature and humidity controlled room with 12 hours (h) light and 12 h darkness before being sacrificed. All experimental manipulations were undertaken in accordance with the National Institutes of Health Guide for the Care and Use of Laboratory Animals, with the approval of the Scientific Investigation Board of the Chinese PLA General Hospital, Beijing, China.

### Intravenous gelsolin infusion

Animals were randomly divided into five groups: intact controls, sham-burn mice, placebo controls that underwent burn injury with an equivalent amount of bovine serum albumin (BSA; Fisher Scientific, Fair Lawn, NJ), and burned mice treated with either a low dose (2 mg/kg, Gsn-L) or a high dose (20 mg/kg, Gsn-H) of recombinant human gelsolin (Sigma-Aldrich, Shanghai, China), according to a previous report [31], in 0.1 ml of sterile saline *via *tail vein immediately after burn injury. Then the animals (9-10 mice per group) were sacrificed at 8, 24, 48 and 72 h postburn (pb). Tissue and plasma samples were collected and stored at -80°C.

### Survival rate

Survival rates were recorded for the low- or high-dose gelsolin-treated mice (n = 30 per group), the placebo-treated mice (n = 30), and the sham-injured mice (n = 10) without further intervention. Differences in survival rates among the groups were analyzed by the Kaplan-Meier method using an SPSS software package.

### Functions of T lymphocytes

Splenic mononuclear cells (MNC) were separated by Ficoll-Paque density centrifugation and were cultivated in complete RPMI-1640 medium in flat-bottomed 96-well microtitre plates (4 × 10^5 ^cells per well) stimulated by the T-cell mitogen concanavalin A (ConA, 5 mg/L; Sigma) for 48 h. Cell-free supernatant fractions were collected and stored at -80°C until analysis for IL-2 by ELISA (ExCell Biology Inc., Shanghai, China). T cell proliferation was examined using a 3-(4, 5-dimethylthiazol -2-yl)- 2, 5-diphenyltetrazolium bromide (MTT) method with absorbance at 450 nm in a multiplate spectrophotometer (Spectra MR; Dynex, Richfield, MN, USA).

### Tissue preparation for immunostaining

Mice (3-4 per group) were killed by cervical dislocation and the brains were removed and post-fixed for 24 h in 4% paraformaldehyde solution, followed by 30% sucrose in phosphate buffer saline (PBS) for another 24-48 h. Brains were stored at -80°C until used to prepare frozen sections at 30 μm thickness. These were serially collected in PBS and finally stored in cryoprotectant solution at -30°C. Some of the brain sections were mounted on lysine-coated slides and stained with hematoxylin and eosin (H&E).

### Quantitative polymerase chain reaction (PCR)

Brains from the remaining mice (5-6 mice per group) were carefully dissected and collected, snap frozen in liquid nitrogen, and stored at -80°C. Different regions (cortex, hippocampus and striatum) were used for total RNA extraction using a NucleoSpin^® ^RNA II Kit (Macherey-Nagel Inc., PA, USA) following the manufacturer's instructions, and used for cDNA synthesis with Superscript II (Promega, Beijing, China). Real-time PCR amplification was achieved in 25 μl reaction mixtures containing 5 μl of cDNA sample, 12.5 μl of SYBR Green PCR Master Mix (SYBR green; Applied Biosystems, Foster City, CA, USA) and specific primers (SBS Genetech Co. Ltd, Beijing, China). An ABI Prism 7700 sequence detection system (Applied Biosystems) with SYBR-green fluorescence was used for assay. Cycling conditions were a 10-min hot start at 95°C followed by 5 cycles of denaturation steps at 95°C for 40 s, an annealing step at 60°C for 30 s, and an extension temperature at 72°C for 30 s. Each sample was run in triplicate. β-actin was used as housekeeping mRNA to normalize gelsolin transcript abundance. Data were analyzed by using sequence Detector Systems version 2.0 software.

Each sample was tested in triplicate. The relative concentration of mRNA was calculated using the formula x = 2^-ΔΔCt^, where x fold change in the target gene at each detection time, normalized to β-actin and relative to the expression of intact mice [32].

### Immunohistochemistry

Sections used for immunocytochemistry were incubated in 0.3% hydrogen peroxide (H_2_O_2_) for 10 min, and incubated free-floating in antibodies (Abs) of polyclonal anti-mouse ionized calcium-binding adapter molecule 1 (Iba-1, 1:1000; Wako, Osaka, Japan), monoclonal anti-mouse CD11b (Mac-1, 1:1000; EuroBioScience, Lund, Sweden), monoclonal anti-mouse CD45 (1:1000; EuroBioScience), or rabbit anti-cleaved caspase-3 (1:50; Cell Signaling, Danvers, MA, USA) with 3% normal goat serum, 0.05%Triton-X in PBS, for 24-48 h rotating at 4°C. The tissue was then rinsed in PBS and incubated for 1 h in biotinylated anti-rabbit IgG (1:200; Vector Laboratories, Burlingame, CA, USA), rotating at room temperature. The tissue was then rinsed in PBS and incubated for 1 h in ABC solution (Vector Laboratories). Following incubation, sections were rinsed with PBS for 20 min and were developed by incubating in 0.025% diamino-benzidine (DAB; Sigma-Aldrich) and 0.002% H_2_O_2 _in PBS. The DAB reaction was halted using PBS, followed by three 10-min PBS rinses.

### Quantification of immunohistochemistry

For quantitative image analysis of periventricular immunostaining, serial sagittal sections of one hemisphere were collected (lateral position +0.5 to +2.25 from Bregma). Iba-1-, CD11b- and CD45-immunostained preparations of sagittal brain sections were evaluated for 4-5 animals from each group. For each animal, antigens were detected in 10 parallel sections having a distance of 70 mm from each other and showing both striatum and cortex. All images were acquired on a BX-61 microscope (Olympus Optical Co., Tokyo, Japan), equipped with a digital camera (F-View II; Olympus Optical Co.). Quantification of immunoreactive cells within the cortex and the striatum was performed at 40 × magnification by a researcher blinded to the treatment. For each animal, average values from all sections were determined.

### Neuron-specific enolase (NSE) and soluble protein-100 (S100) detection

Brain tissues were weighed and homogenized after addition of 3 ml/g (1:4) saline with protease inhibitor cocktail (Applygen Technologies Inc., Beijing, China). The supernatants were collected for NSE and S100 analysis in duplicate using available quantitative 'sandwich' enzyme-linked immunosorbent assay kits (Rapidbio, CA, USA). Sensitivity of the assays was 1.0 pg/ml for S100 and 0.1 ng/ml for NSE.

### Western blot

The dissected brain tissues were collected, snap-frozen in liquid nitrogen and stored at -80°C. Tissue was homogenized in RIPA buffer with protease inhibitor (Applygen Technologies Inc.). The total amount of protein was determined by bicinchoninic acid protein assay (Applygen Technologies Inc.). Samples (100 μg protein) were separated by 8% SDS-PAGE and electroblotted to nitrocellulose membrane, which were blocked by incubation in 3% (w/v) bovine serum albumin dissolved in TBS-T (150 mM NaCl, 50 mM Tris, 0.05% Tween 20). Following transfer, proteins were probed using a rabbit monoclonal phospho-p44/42 extracellular regulated kinase 1/2 (ERK1/2) (1:2000; Cell Signaling) in TBS-T. Horseradish peroxidase-conjugated secondary Ab was used at a 1:1000 dilution in TBS-T. After extensive washing, protein bands detected by Abs were visualized by ECL reagent (Applygen Technologies Inc.) after exposure on autoradiograph film (Fuji Film; Kodak Scientific Imaging Film, Beijing). Membranes were then stripped and re-probed with p44/42 MAPK (ERK1/2) mouse monoclonal Ab (1:1000; Cell Signaling) to confirm equal protein loading. The films were subsequently scanned, and band intensities were quantified using Image software.

### Assessment of cysteinyl aspartate-specific protease (caspase)-3 activity

Caspase-3 activity was measured using a colorimetric assay according to the manufacturer's instructions (BioVision, Mountain View, CA, USA). The brain tissues were lysed in buffer (50 mM HEPES, pH 7.4, 0.1% CHAPS, 1 mM DTT, 0.1 mM EDTA and 0.1% Triton X-100) and centrifuged at 12, 000 × *g *for 10 min at 4°C. After determination of protein concentration by bicinchoninic acid method (Applygen Technologies Inc.), the cell extract (200 μg of protein) was added to the assay buffer (100 mM HEPES, pH 7.4, 0.1% CHAPS, 10 mM DTT, 10% glycerol, and 2% (v/v) dimethylsulfoxide) containing chromogenic substrates (2 mM) and incubated for 4 h at 37°C. Caspase-3 activity was determined by measuring the absorbance at 405 nm using a microplate reader (Spectra MR; Dynex, Richfield, MN, USA).

### Determination of plasma gelsolin concentrations

At 8, 24, 48 and 72 h after burns or sham injury, the animals were anesthetized, and blood obtained by cardiac puncture was placed in a heparinized tube (n = 6 samples each group per time point). The blood was centrifuged and plasma gelsolin concentrations were determined in duplicate with a mouse gelsolin ELISA detection kit (USCN Life, Wuhan, China).

### Statistic analysis

All data are expressed as mean ± SD from three or more independent experiments. Statistical comparisons among different groups were done by one-way analysis of variance (ANOVA) with Dunnett's multiple comparison tests using SPSS software (IBM, Beijing, China). Differences with p < 0.05 were considered statistically significant.

## Results

### Administration of gelsolin can improve the survival rate of burn mice

**Figure 1:**
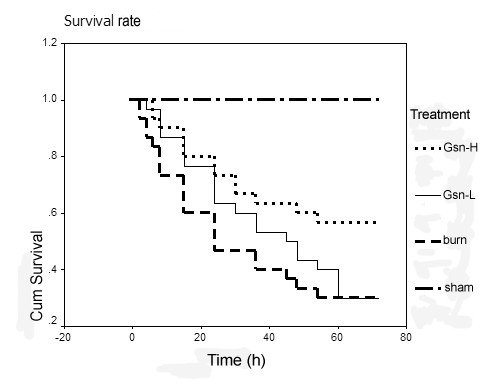
**Survival rates in burn-injured mice after treatment with exogenous gelsolin at low (Gsn-L) or high dose (Gsn-H)**. There was greater mortality for placebo (burn, 21 of 30) than for Gsn-H-treated (13 of 30) mice after thermal injury, and survival time was significantly shorter in placebo-injected mice than in Gsn-L or Gsn-H-treated mice.

The survival of gelsolin-treated mice at low (Gsn-L) or high doses (Gsn-H), as well as of placebo-treated mice, was assessed over a 168 h period after burn injury (Figure 1). All the mice exposed to sham injury survived the entire period (n = 10). Placebo-treated mice had a higher mortality than Gsn-H mice (70% versus 43.33%, p < 0.05) within 72 h after burn injury, and no further mortality occurred after that observation period. Mean survival time was prolonged in the Gsn-H group (51.17 ± 4.7 h, p = 0.0258 versus placebo) and the Gsn-L group (43.13 ± 4.46 h, p = 0.4875 versus placebo) in comparison with the placebo group (35.5 ± 4.96 h). Nevertheless, there was no significant difference in mean survival time between Gsn-L and Gsn-H groups (P = 0.0791).

### Treatment with gelsolin obviously ameliorated burn-induced brain damage

**Figure 2:**
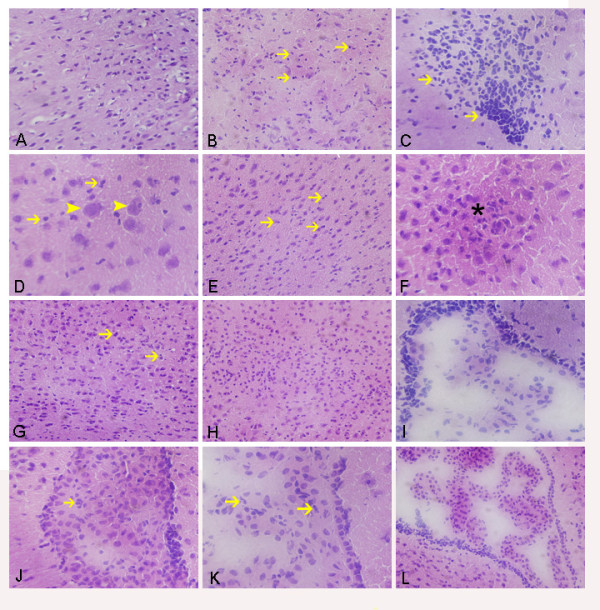
**Representative images of H&E-stained sections, highlighting cerebral sparing by high dose gelsolin in burn-injured mice**. Cortex of control mice (A) and burned mice at 8 h postburn (B, C, D), 24 h postburn (E, F) and 24 h following low dose (G) and high dose (H) gelsolin treatment. Images of the lateral ventricles, including the choroid plexus, at 8 h (I), 24 h (J) postburn, and 24 h after gelsolin treatments at low dose (K) and high dose (L) respectively. →: leukocyte, ➤: neurosis, *: microabscess.

As compared with sham-injured mice (Figure 2A), the brains of mice subjected to thermal injury exhibited typical pathological lesions. There was invasion of dispersed, or even clustered leukocytes in the cortex (Figure 2B) and the striatum (Figure 2C) as early as 8 h pb. Concurrently, neurons were shrunken with condensed nuclei, suggesting an early stage of apoptosis (Figure 2D). As late as 24 h pb, a dispersed infiltration of leukocytes (Figure 2E) and even microabscesses (Figure 2F) were seen in the cortex of the mice, indicating a progressive infiltration of inflammatory cells in brain over this time period. At 24 h pb, dispersed leukocytes were still observed in the cortex of Gsn-L mice, suggesting that treatment with gelsolin at low dose fails to ameliorate the burn-induced brain injury (Figure 2G). In contrast, administration of gelsolin at high dose could protect the brain from undergoing the pathological changes described above (Figure 2H). Similar results were also obtained for Gsn-H mice at other time points (data not shown).

Strikingly, the leukocyte infiltration occurred in the ependymal layer of the lateral ventricle as early as 8 h pb (Figure 2I). In the worst situation, the lateral ventricle was filled with inflammatory exudates at 24 h pb (Figure 2J). Moreover, a few leukocytes were observed to accumulate in the choroid plexus in brain of Gsn-L mice (Figure 2K), but seldom in Gsn-H mice (Figure 2L) at 24 h pb.

**Figure 3:**
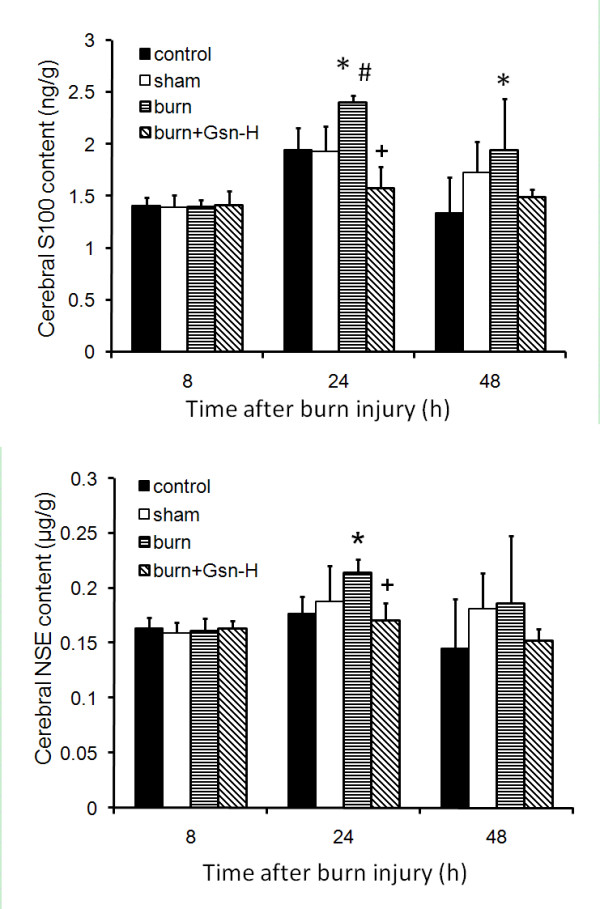
**Gelsolin at high dose (Gsn-H) reduces brain-specific proteins, S100 (A) and NSE (B), in mice following burn injury**. All data are expressed as mean ± SD of the mean (n = 6). *P < 0.05 vs. intact control, #p < 0.05 vs. sham-injured, +p < 0.05 vs. placebo mice.

Consistent with the morphological observations, the neural injury markers cerebral S100 and NSE content were reduced by high dose gelsolin treatment at 24 h pb, while these remained at levels similar to control or sham-burned mice at 8 h pb (Figure 3). It is noteworthy that both S100 and NSE showed a small trend of increase at 48 h pb, which could also be slightly reduced by gelsolin infusion at high dose.

### Treatment with gelsolin decreased burn-induced proinflammatory cytokines in the brain

To further validate and explore the above findings, we next investigated the time course of mRNA expression of proinflammatory cytokines by real-time PCR in brain of burned mice. On account of the lack of significant improvement in pathology in Gsn-L mice, only the gene expression of proinflammatory cytokines in the brains of Gsn-H mice was determined.

**Figure 4:**
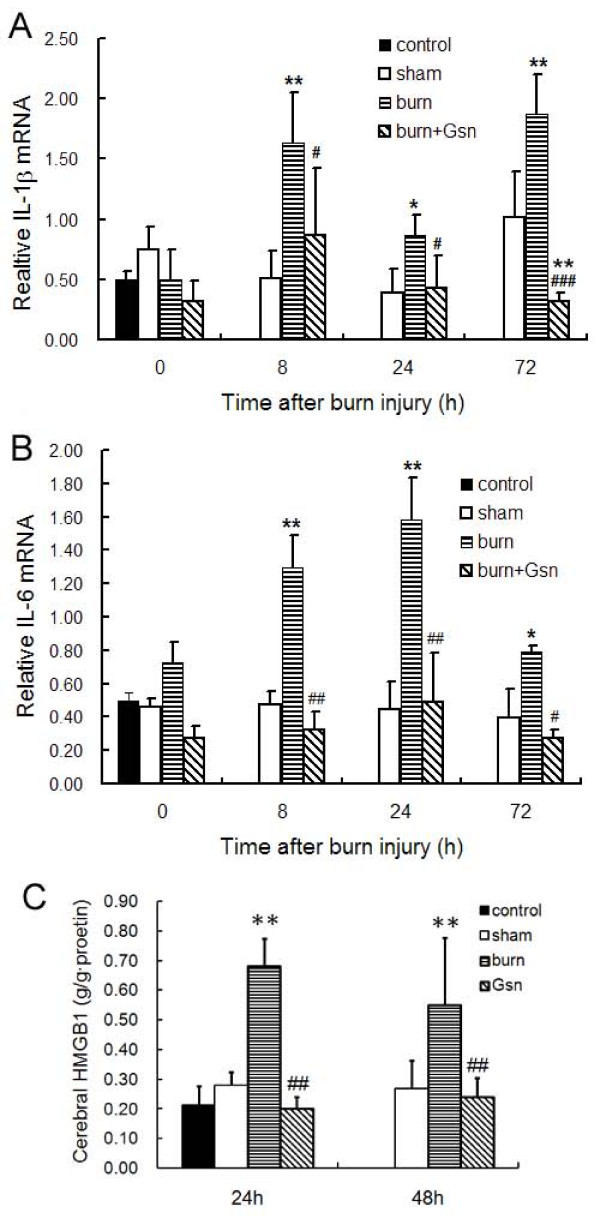
**Gelsolin administration protects against burn-induced proinflammatory cytokine expression in brain**. Elevated levels of IL-1β (A) and IL-6 (B) mRNA, as well as HMGB1 content (C) were found in cortex after burn injury. Data are shown as mean ± SD for n = 6. *P < 0.05 and **P < 0.01 vs. sham-injured mice; #p < 0.05, ##p < 0.01, ### p < 0.001 vs. placebo mice; +p < 0.05 and ++p < 0.01 vs. Gsn-L by ANOVA, Newman-Keuls post-hoc test.

Significant reductions in brain levels of early cytokines, including IL-1β and IL-6 mRNA expression, and late cytokine high mobility group box-1 protein (HMGB1), were found in the gelsolin-treated group compared to the placebo group at all time points (Figure 4). Most strikingly, IL-1β mRNA expression in the placebo mice spiked rapidly, and continued to increase at various time points (Figure 4A). IL-6 mRNA expression in brain tissue was increased by approximately 1.5- to 2-fold that of the placebo group compared to normal controls following thermal injury (Figure 4B). Gelsolin injection resulted in marked down-regulation of IL-1β mRNA expression compared with the placebo group. Similarly, IL-6 mRNA levels in the brain were suppressed by approximately 70% in the gelsolin-treated group compared with the placebo group, close to that of the sham-injured group (Figure 4B).

HMGB1 is a non-histone DNA binding protein that is secreted by activated monocytes and macrophages [33], and passively released by necrotic or damaged tissues [33-35] including brain [36]. Thus, HMGB1 acts as an immediate trigger of inflammation [37] as well as a late mediator of inflammation [33]. We found that HMGB1 levels were significantly elevated in the brain at 24 and 48 h pb, while they were markedly decreased by gelsolin treatment at both dosages (Figure 4C).

In addition, we did not find changes in IL-17A or IL-10 mRNA in the brain tissue, implying that there might be no T cell infiltration in brain secondary to acute burns. Similarly, there was no expression of anti-inflammatory cytokines, including IL-10 mRNA, induced by gelsolin infusion (data not shown).

### Administration of gelsolin suppressed burn-induced microglial activation in the brain

Microgliosis is a common feature of central nervous system (CNS) injury and disease, and this involves microglial cell division, hypertrophy, and alterations in immunophenotype as well as secretory activity [38]. The augmented neuroinflammation may lead to dysregulation of microglial number and/or microglial activation in the CNS. To test such a hypothesis, we stained microglial populations in brains of burned mice with the microglial marker (Iba-1) at different intervals.

**Figure 5:**
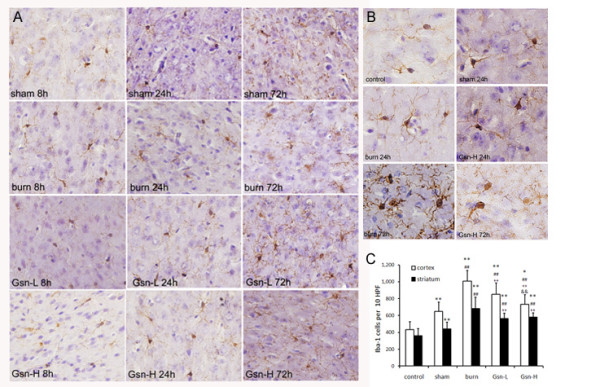
**Treatment with gelsolin reduces microglial activation, as assessed by ionized calcium binding adaptor molecule 1 (Iba-1) expression after burn-induced neuroinflammatory responses in the cortex 24 h postburn shown at low (A, × 200) and high (B, × 400) magnifications**. Cell counting in cortex and striatum was performed to show that the increased Iba-1 levels after thermal injury were suppressed by a high dose of gelsolin 72 h postburn (C). All pictures are representative of brain sections from 3 mice for each time point. *P < 0.05 and **P < 0.01 vs. sham-injured mice; ##p < 0.01 vs. placebo mice; ++p < 0.01 vs. Gsn-L mice by ANOVA, Newman-Keuls post-hoc test. Data are means ± SD for n = 6.

We observed kinetic changes of Iba-1-immunoreactive cells in striatum and cortex after thermal injury. In brief, Iba-1-immunoreactive cells showed morphological changes and altered immunoreactivity in cortex, striatum and CA1 region with time after acute insults, peaking at 72 h pb in the striatum region (Figure 5A). Iba-1^+ ^cells were well ramified in burned mice in contrast with a highly ramified 'resting' morphology in sham-injured brain (Figure 5B). These alterations might be associated with delayed neuronal death of striatum cells in burned mice. In contrast, gelsolin administration at either dosage could suppress activation of Iba-1^+ ^microglia in cortex and striatum as exemplified by mice at 72 h pb, correlating with its anti-inflammatory effect in brain (Figure 5C).

Taken together, immunohistochemistry analyses revealed enhanced microglial density and activation status in brain at 72 h pb, implicating delayed activation of microglial proliferation and/or activation responses after thermal injury.

### Caspase-3 activation in the brain was inhibited by gelsolin infusion after burn injury

**Figure 6:**
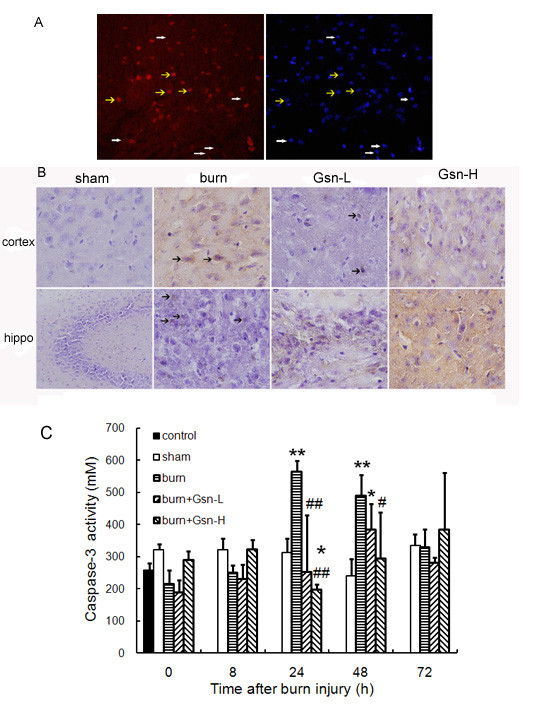
**Gelsolin decreases caspase-3 activities in brain of mice following burn injury**. A. Immunofluorescent staining of caspase-3 in striatum of brain 24 h postburn; B. Immunohistochemistry of caspase-3 positive cells in the cortex and hippocampus (hippo) from mice under gelsolin treatments. C. Time course of caspase-3 activity in brain as assayed by colorimetry. *P < 0.05 and **P < 0.01 vs. sham-injured mice; #p < 0.05 and ##p < 0.01 vs. placebo mice by ANOVA, Newman-Keuls post-hoc test. Data are means ± SD for n = 6-8.

Caspase-3-positive cells were detected in striatum of burned mice by immunofluorescence (Figure 6A). Immunohistochemistry analysis also verified reduced caspase-3-positive cells in both cortex and hippocampus by gelsolin treatment (Figure 6B). To determine if gelsolin could inhibit caspase-3 activation in our model, we measured levels of caspase-3 activity in the brain tissue. We found that there was an approximately 2-fold increase in caspase-3 activity in the placebo group in comparison to the sham group at 24 h and 48 h pb. However, at early 8 h and later 72 h time points, there were no marked differences in caspase-3 activity between the placebo and sham groups. As expected, gelsolin injection either at low or high dosage could reduce the elevated caspase-3 activity to levels comparable to sham-injured mice at 24 h pb, while at later time points such as 48 h pb, only the high dose of gelsolin could exert a similar effect (Figure 6C).

### Gelsolin enhanced CD11b and CD45 monocyte/macrophage recruitment into brain following burn injury

**Figure 7:**
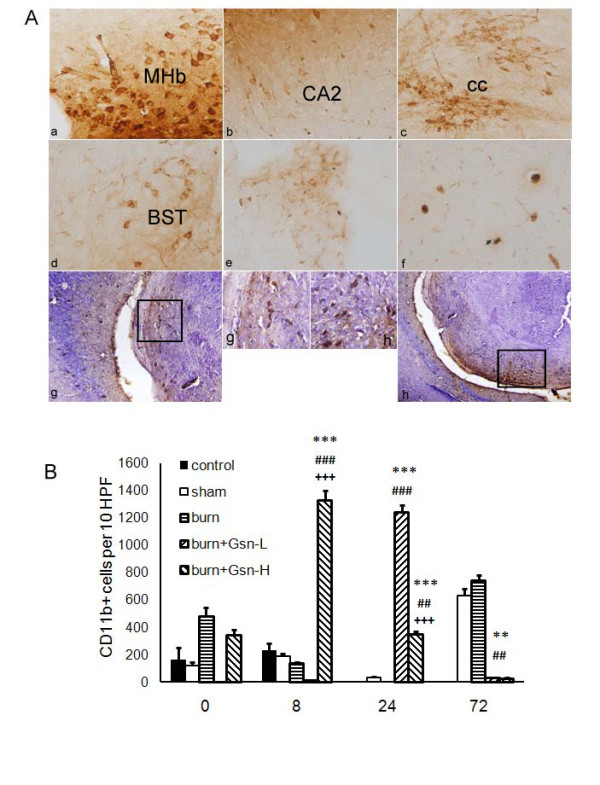
**Gelsolin affects migration of myeloid-derived cells into brain**. CD11b+ cells from mice 72 h postburn (A) and quantification of infiltrating CD11b+ (B) cells in 10 high power fields (HPF) of the periventricle region following gelsolin treatment. Gelsolin positive cells were seen in medial habenular nucleus (MHb, a), hippocampal CA field (CA2, b), corpus callosum (cc, c), bed nucleus striatum terminal (BST, d), choroid plexus (e), cortex (f), lateral ventricle (g, h) and amplified lateral ventricle (g', h'). Magnifications for "a-f" and "g'-h'" are × 400, "g-h" are × 200. *P < 0.05, **P < 0.01, and ***P < 0.001 vs. sham-injured mice; #p < 0.05, ## p < 0.01, ### p < 0.001 vs. placebo mice; ++p < 0.01, and +++p < 0.001 vs. Gsn-L mice by ANOVA, Newman-Keuls post-hoc test. Data are means ± SD for n = 6-8.

**Figure 8:**
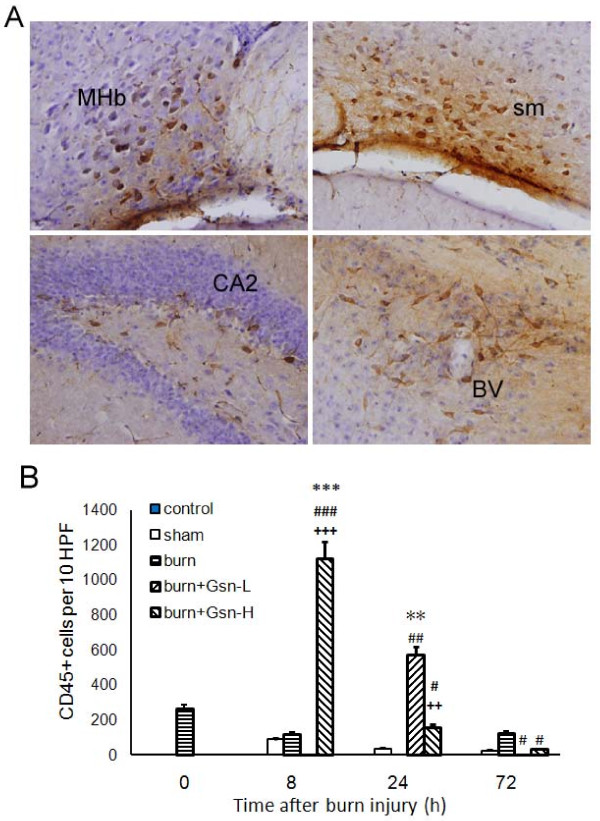
**Gelsolin affects migration of myeloid-derived cells into brain**. CD45^+ ^cells from gelsolin-treated mice 8 h postburn (A) and quantification of infiltrating CD45^+ ^(B) cells in 10 high power fields (HPF) of the periventricle region following gelsolin treatment. Gelsolin positive cells were seen in medial habenular nucleus (MHb), stria medullaris (sm), hippocampal CA field (CA2) and blood vessel (BV). Magnifications are × 400. *P < 0.05, **P < 0.01, and ***P < 0.001 vs. sham-injured mice; #p < 0.05, ##p < 0.01, ###p < 0.001 vs. placebo mice; ++p < 0.01, and +++p < 0.001 vs. Gsn-L mice by ANOVA, Newman-Keuls post-hoc test. Data are means ± SD for n = 6-8.

CD11b is expressed by mature monocytes [16] and by monocyte-derived microglia-like cells [39], and CD45 is a pan-leukocyte marker. Unexpectedly, an increase in absolute numbers of macrophage/microglial cells (CD11b^+^CD45^+^) [40] was found in the gelsolin-treated groups. It is intriguing that numerous CD11b^+ ^infiltrating monocytes and resident microglial cells with prominent amoeboid morphology were noted in the periventricular regions at 24 h pb (Figure 7A). This morphology is generally associated with activated microglia or macrophages. Since gelsolin is known as a strong chemoattractant [22], we further investigated the involvement of gelsolin in the migration of myeloid-origin cells into the brain. To our surprise, the numbers of CD11b^+ ^cells were increased in sham and burn-injured groups as late as 72 h pb, implying that activation of CD11b^+ ^cells was delayed. By contrast, the number of CD11b^+ ^cells was decreased by treatment with gelsolin at both dosages (Figure 7B). However, CD45^+ ^macrophages accumulated in the perivascular regions at 8 h pb in the Gsn-H group (Figure 8A). At both 8 h and 24 h pb, numbers of CD45^+ ^cells were arrested in the periventricular region by both doses of gelsolin administration with differential effects (Figure 8B).

### Gelsolin down-regulated burn-mediated ERK1/2 phosphorylation in brain

**Figure 9:**
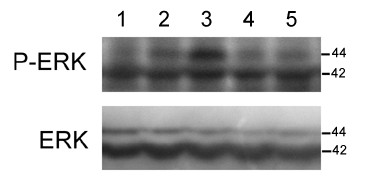
**Gelsolin down-regulates burn-induced phospho-ERK1/2 activity in hippocampus**. Lane 1: sham control, Lane 2: 8 h postburn, Lane 3: 24 h postburn, Lanes 4-5: Gsn-H 24 h postburn. The ERK1/2 blotting displayed equal loading between wells.

Western immunoblotting for the active, dually phosphorylated form of p44/42 mitogen-activated protein kinase (MAPK) (ERK1/2) revealed that thermal injury *per se *resulted in activation of this signal pathway in brain tissue (Figure 9). An increase in phosphorylation of p44/42 MAPK was observed at 8 h pb, and this was remarkably increased at 24 h pb. ERK1 (44 kDa) density of the sham group was 18, 207 ± 829, while it reached 25, 564 ± 914 and 30, 546 ± 1077 in burned mice at 8 h and 24 h, respectively (P < 0.05). Exogenous infusion of gelsolin could markedly down-regulate ERK1/2 phosphorylation at 24 h pb (12, 883 ± 877).

### Gelsolin improved the suppressed T lymphocytes functions induced by burn injury

**Figure 10:**
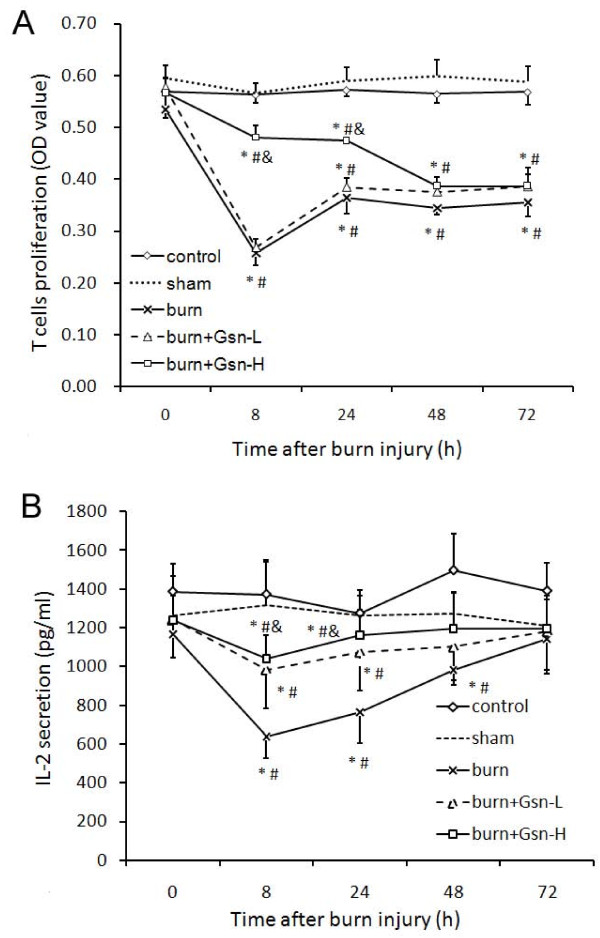
**Gelsolin restores suppressed T lymphocyte function after burn injury**. Splenic mononuclear cells harvested from the mice under different treatments were cultured in the presence of the T-cell mitogen concanavalin A (5 mg/L) for 48 h. T-cell viability was determined by MTT methods (A) and the supernatants were collected for IL-2 analysis (B). Data are shown as mean ± SD for n = 6-8. *P < 0.05 vs. intact control; #p < 0.05 vs. sham-injured mice; &p < 0.05 vs. placebo mice by ANOVA, Newman-Keuls post-hoc test.

As expected, burn injury resulted in dramatic suppression of T cell function, as shown by decreased proliferation (Figure 10A) and IL-2 secretion (Figure 10B), compared with either intact control or sham-burned mice. Although infusion of gelsolin at high dose could partially prevent the decline, gelsolin at low dose failed to exert any effect on T cell function.

### Kinetic changes in plasma gelsolin concentrations

To evaluate kinetic changes in circulating gelsolin in various groups in the study, we measured plasma gelsolin concentrations to determine its bioavailability. We found that in animals receiving gelsolin, there were not dramatic changes in circulating gelsolin concentrations. As exemplified in the Gsn-H group, gelsolin concentrations ranged from 851 ± 32 pg/ml at 8 h pb to 844 ± 128 pg/ml at 72 h pb. Baseline values of plasma gelsolin in both the intact control and placebo groups was approximately 1300 pg/ml, and they remained relatively constant at all observed time points (Table 1). Following burn injury, plasma gelsolin levels dropped rapidly to only 1/3 of the sham group within 8 h (407 ± 57 versus 1273 ± 145, P < 0.001) and remained at low levels for up to 3 days. Although plasma gelsolin levels in the Gsn-H group were almost twice that of the placebo group at most time points, gelsolin administration in both dosages could only prevent part of the reduction in plasma gelsolin levels that accompanied burn injury compared with the sham-burned group.

**Table 1:** Circulating levels of gelsolin (pg/ml) after exogenous gelsolin administration in mice after burn injury.

group	0 h	8 h	24 h	48 h	72 h
control	1294 ± 113	1295 ± 81	1276 ± 77	1297 ± 69	1229 ± 50
sham	1295 ± 111	1273 ± 145	1284 ± 96	1290 ± 135	1292 ± 68
burn+placebo	1136 ± 52	407 ± 57*	484 ± 117*	559 ± 106*	529 ± 169*
burn+Gsn-L	1216 ± 52	662 ± 67*^#^	862 ± 67*^#^	803 ± 197*^#^	817 ± 125*^#^
burn+Gsn-H	1193 ± 104	851 ± 32*^#+^	923 ± 63*^#^	956 ± 87*^#+^	844 ± 128*^#^

*P < 0.05 vs. the sham-injured group; #p < 0.05 vs. the placebo group; +p < 0.05 vs. the Gsn-L group by ANOVA, Newman-Keuls post-hoc test. Data are means ± SD for n = 6-8.

## Discussion

A successful therapeutic strategy for brain injury should include inhibition of proinflammatory cytokines, promotion of anti-inflammatory cytokines, suppression of autoimmunity to CNS antigens and reduction in recruitment of inflammatory cells, etc. In the present study, we report protective effects of gelsolin in brain of mice subjected to burn injury, characterized by amelioration of pathological lesions and suppression of microglial activation, which might be associated with enhanced recruitment of CD11b^+ ^as well as CD45^+ ^cells. Likewise, gelsolin could substantially down-regulate the marked expression of both early (IL-1β, IL-6) and late proinflammatory cytokines (HMGB1) in the brain. In addition, treatment with gelsolin significantly reduced caspase-3 activity and inhibited ERK phosphorylation in the brain secondary to severe burns.

As a 90 kDa protein, it is not likely that gelsolin easily penetrates into brain to perform its effects. Yet a pioneer study has demonstrated that peripherally expressed plasma gelsolin can affect amyloid-*β *dynamics in the CNS in two mouse models of Alzheimer's disease (AD) [41]. The authors suggested that one possible clearance mechanism might be *via *plasma gelsolin entrance into brain parenchyma across the BBB, as reports have indicated that the BBB is compromised in mouse models of AD [42]. Similarly, an increase in the permeability of the BBB is a common event in thermally injured animals [8], as also shown in our study by the filling of the lateral ventricles with inflammatory exudates, so it is reasonable to speculate that intravenous infusion of gelsolin could penetrate the BBB into brain parenchyma to attenuate neuroinflammation.

Inflammatory mediators are able to alter cellular metabolism by inducing oxidative stress and mitochondrial dysfunction [43], resulting in pathologic abnormalities [44]. Abnormally high levels of cytokines in brain have been found to correlate with both morbidity and mortality in patients with extensive burn injury. In our study, cerebral IL-1β and IL-6 mRNA were up-regulated around 8 h pb and kept increasing throughout the entire period. It is likely that HMGB1 levels were significantly elevated in brain at both 24 and 48 h pb. Gelsolin treatment could significantly reduce expression and release of early as well as late proinflammatory cytokines. This down-regulation of the inflammatory response would lead to less damage and cell loss in the brain, which might, in the future, allow preservation of cognition in patients with severe burn injury. IL-10 is an immuno-suppressant that is mainly secreted by regulatory T cells. It is well known for its positive effects in cerebral ischemia in rats [45]. We did not detect IL-10 gene expression in brain during the entire observation period, suggesting that administration of gelsolin only inhibits proinflammatory cytokine transcription, without augmenting expression of anti-inflammatory cytokines.

Microglial cells are the primary immune effector cells in brain and play a pivotal role in neuroinflammatory processes associated with a variety of neurological as well as pathological disorders. Microgliosis is a common feature of CNS injury and disease [38]. Iba-1 is specifically expressed in microglia and plays an important role in regulation of microglial function. Increased Iba-1 immunoreactivity is a hallmark of burn-induced inflammation. It has been proposed that microglial activation induced by sepsis is involved in the pathogenesis of delirium [46]. There are no reports to date dealing with direct investigation of the activation of microglia and glial scarring following severe burns. We observed kinetic changes in Iba-1^+ ^cells in striatum and cortex after thermal injury, indicating a highest level of activation as late as 72 h pb. A number of studies have demonstrated that activated glial cells participate in the degeneration of dopamine neurons [47]. Our data suggest that burn injury *per se *might result in microgliosis and loss of vulnerable neuronal populations from inflammation-induced cell death.

Inflammation and apoptosis are two of the most important underlying causes of septic encephalopathy [48]. Because local accumulation of cytokines may induce apoptosis and significantly extend the initial injury, we also wanted to clarify whether the ability of gelsolin to down-regulate cytokine signaling could lead to decreased activation of apoptotic proteins in brain. Previous investigations have documented that severe burn injury is associated with a significant increase in apoptosis in remote organs [30,49,50] including brain [47]. A number of markers such as S100B and NSE can serve as general markers of brain injury. Consistent with our observation of morphological improvement, cerebral S100B and NSE levels were diminished by gelsolin infusion. Our study further proves that gelsolin administration immediately following burn injury can reduce caspase-3 activity in brain, confirming a neuroprotective effect of gelsolin.

In inflammatory diseases either inside or outside the CNS, communication between the periphery and the brain *via *humoral and/or neural routes results in central neural changes and related behavioral alterations. Monocytes are circulating antigen-presenting leukocytes that play critical roles in inflammation, T-cell differentiation, phagocytosis, and innate immunity [51,52]. Previous studies have reported significant infiltration of activated monocytes into the brain of mice with hepatic inflammation [18], stroke [19], ischemia-reperfusion [15] and bacterial meningitis during the post-inflammatory period [16]. Importantly, these newly recruited monocytes became an integral part of the pool of parenchyma microglia and contribute to the clearance of damaged tissue [17]. CD11b is expressed by mature monocytes [16] and monocyte-derived microglia-like cells [39], whereas CD45 is a pan-leukocyte marker. Resolution of CNS infection is often the result of a balance between immune-mediated pathogen clearance and the deleterious effects of inflammation. Indeed, in a murine model of rabies encephalitis, administration of a sex steroid enhanced permeability of the BBB, promoted immune cell penetration into the CNS, and improved survival [53]. It has also been reported that gelsolin is necessary for rapid motile responses in cell types involved in stress responses such as hemostasis, inflammation and wound healing [54]. In gelsolin-mutant mice, macrophage motility was impaired and this contributes to a reduced inflammatory response [54] and a reduced capacity to recruit macrophages to the injury site, which in turn, slows the clearance of myelin debris and consequently remyelination [22]. Consistent with these findings, we noticed that gelsolin infusion could accelerate the recruitment of CD11b^+ ^and CD45^+ ^cells into the periventricular region of brain early after burn injury, but could still exert a suppressive effect on their recruitment at 72 h pb, indicating an early recruitment of monocyte/macrophage by gelsolin. The increased penetration of CD11b+ cells and the enhanced microglial activation in gelsolin-treated animals were found to be associated with down-regulation of proinflammatory cytokines and caspase-3 activities. Taken together, these results indicate that treatment with gelsolin could ameliorate inflammatory responses in brain and apoptosis of cerebral cells after burn injury.

To elucidate the potential signaling mechanism underlying gelsolin-mediated neuroprotective activity, we examined expression levels of phospho-ERK in burn mice. Western blotting experiments using anti-phospho-p44/42 MAPK (ERK1/2) mouse antibodies revealed activation of phospho-ERK in brain following thermal injury, which is consistent with previous reports [7,13]. ERK activation may be downstream of free radicals formation, based on the finding that dopaminergic cells exposed to 6-hydroxydopamine, a reactive oxygen species generating neurotoxin, exhibit a distinct temporal pattern of ERK1/2 activation and caspase-3 activity [55]. It has been demonstrated that neurons are damaged following prolonged exposure to high concentrations of corticosterone, with activation of p38 MAPK, ERK1/2, and c-jun N-terminal protein kinase 1 [56], particularly in chronic inflammatory and immune diseases. The increased phospho-ERK levels in brain following burn injury might be a consequence of multiple factors, including proinflammatory mediators, ischemia, and oxidative stress.

We further found that gelsolin treatment dramatically inhibits expression of phospho-ERK1/2 in brain of burned mice. These biochemical results are not in agreement with a previous observation that the neuroprotective effects of estrogen could be attributed to increased phospho-ERK in brain [13]. However, other authors have reported that administration of neuroprotective reagents reduces phospho-ERK1/2 activity [57,58], and inhibition of ERK1/2 can protect against brain damage resulting from focal cerebral ischemia [59]. Furthermore, it has been demonstrated that gelsolin overexpression inhibits ERK1/2 phosphorylation, nuclear factor of activated T-cell activation, and IL-2 production [23]. Thus, the exogenous supply of gelsolin in our experiments might protect the brain from exposure to pro-apoptotic stimuli, which in turn might down-regulate ERK1/2 phosphorylation.

Although cerebral complications have been related to increased mortality in severe sepsis [60] and major burn victims [10], attenuation of neuroinflammation might not account for all of the benefit of gelsolin in reducing burn-induced mortality in our study. Considering that burn injury could result in severe suppression of the immune system, which plays an important role in the development of subsequent sepsis, multiple organ failure and even death, we examined the dynamic changes in immune function of splenic T cells as well. We found an immunosuppressive effect involving T cells following burn injury, which is consistent with a previous report of perturbed T cell homeostasis after burn injury [61]. It was encouraging to find that gelsolin infusion could markedly enhance cell-mediated immunity of splenic T cells, which might also contribute to reduced post-burn mortality.

While these studies are intriguing, there are several limitations that should be addressed in future investigations. The first shortcoming is that clinical outcome variables were not obtained. For clinical relevance, multi-organ dysfunction which may be the root cause of burn-induced mortality should be evaluated in further studies. Secondly, neurological outcomes like edema, BBB penetrability and cognitive function were not assessed in the current study. Better understanding of the improvement of neurological outcome with gelsolin may allow an in-depth understanding of the mechanisms by which gelsolin attenuates the acute response, and to what extent neurological damage contributes to post-burn mortality. Although we initiated this study to observe the acute effects of gelsolin on neuroinflammation following burn injury, it may be possible to solidify our current observations in a further study by also evaluating the effects of gelsolin on these neurological complications which are frequently seen in our clinical patients. Thirdly, with regard to the time-window of gelsolin delivery, intravenous infusion of gelsolin immediately after burn injury resulted in significantly reduced mortality. However, the interventional time could be postponed to later intervals to more closely simulate a clinical setting for this therapeutic strategy. The final, but foremost concern regards the pharmacokinetics of gelsolin in this model. With a half life as long as 2.3 days [62], a single administration of gelsolin could produce considerably elevated gelsolin levels as early as 8 h and remained high at 72 h pb. As BBB disruption may occur as early as 7 h after burn injury [8], while gelsolin might not penetrate the BBB directly within the first hours, and it is reasonable to speculate that gelsolin could breach the BBB to perform its effect directly in the brain at later time points. Nevertheless, the precise mechanism of our observed gelsolin effect on response to thermal injury and immunomodulation in both the brain and the periphery requires further studies.

## Conclusion

Despite these limitations, we conclude that, following severe burn injury in a rodent model, an early, single dose of gelsolin can dramatically reduce mortality by ameliorating cerebral inflammatory lesions and apoptosis *via *acceleration of recruitment of monocytes and down-regulation of phospho-ERK1/2 expression, and also *via *improvement of peripheral T cell functions as well. Although further studies are warranted, these findings might be of importance in the near future in the development of a safe and effective new therapy aimed at significantly improving the outcome of patients with severe burns.

## Abbreviations

Gsn: gelsolin; TBSA: total body surface area; pb: post burn; caspase-3: cysteinyl aspartate-specific protease (caspase)-3; BSA: bovine serum albumin; H&E: hematoxylin and eosin; H_2_O_2_: hydrogen peroxide; Abs: antibodies; DAB: diamino-benzidine; Iba-1: ionized calcium-binding adapter molecule 1; RIPA: radio-immunoprecipitation assay, BCA: bicinchoninic acid; CNS: central nervous system; BBB: blood brain barrier; MAPK: mitogen activated protein kinase; ERK1/2: extracellular regulated kinase 1/2; kDa: kilo Dalton; MTT: 3-(4, 5-dimethylthiazol-2-yl)-2, 5-diphenyltetrazolium bromide; JNK1: c-jun N-terminal protein kinase 1; NF-AT: nuclear factor of activated T-cells.

## Competing interests

The authors declare that they have no competing interests.

## Authors' contributions

QHZ participated in the design of the study; personally conducted a significant portion of the experiments presented in the manuscript, and participated in the writing of the manuscript. QC participated in the design of the study and the preparation of the animal model. JRK prepared all the cryostat sections. LC and XMZ did the cell counting of the brain. ND conducted the QPCR detection. ZYS supervised and edited the manuscript. YMY participated in the design of the experiments, funding of the projects, and preparation of the manuscript. All authors have read and approved the final version of the manuscript.

## References

[B1] Zhou H Andonegui G Wong CH Kubes P Role of endothelial TLR4 for neutrophil recruitment into central nervous system microvessels in systemic inflammation J Immunol 2009 183 5244 5250 10.4049/jimmunol.0901309 19786543

[B2] Mora AG Ritenour AE Wade CE Holcomb JB Blackbourne LH Gaylord KM Posttraumatic stress disorder in combat casualties with burns sustaining primary blast and concussive injuries J Trauma 2009 66 S178 185 10.1097/TA.0b013e31819ce2d6 19359963

[B3] Rosenberg M Robertson C Murphy KD Rosenberg L Mlcak R Robert RS Herndon DN Meyer WJ Neuropsychological outcomes of pediatric burn patients who sustained hypoxic episodes Burns 2005 31 883 889 10.1016/j.burns.2005.05.004 16006044

[B4] Wollgarten-Hadamek I Hohmeister J Demirakca S Zohsel K Flor H Hermann C Do burn injuries during infancy affect pain and sensory sensitivity in later childhood? Pain 2009 141 165 172 10.1016/j.pain.2008.11.008 19095356

[B5] Li H Ying D Sun J Bian X Zhang Y He B Comparative observation with MRI and pathology of brain edema at the early stage of severe burn Chin J Traumatol 2001 4 226 230 11835738

[B6] Li HT Ying DJ He XC Sun JS Chen L Stereoscopic study on capillary density of early brain oedema in a dog postburn model Injury 2009 40 835 839 10.1016/j.injury.2008.10.009 19232588

[B7] Zhang Q Carter EA Ma B Fischman AJ Tompkins RG Burn-related metabolic and signaling changes in rat brain J Burn Care Res 2008 29 346 352 10.1097/BCR.0b013e3181667387 18354292

[B8] Patel TH Sprague S Lai Q Jimenez DF Barone CM Ding Y Blood brain barrier (BBB) dysfunction associated with increased expression of tissue and urokinase plasminogen activators following peripheral thermal injury Neurosci Lett 2008 444 222 226 10.1016/j.neulet.2008.08.020 18718505

[B9] Reyes R Guo M Swann K Shetgeri SU Sprague SM Jimenez DF Barone CM Ding Y Role of tumor necrosis factor-alpha and matrix metalloproteinase-9 in blood-brain barrier disruption after peripheral thermal injury in rats J Neurosurg 2009 110 1218 1226 10.3171/2008.8.JNS08382 19199470

[B10] Flierl MA Stahel PF Touban BM Beauchamp KM Morgan SJ Smith WR Ipaktchi KR Bench-to-bedside review: Burn-induced cerebral inflammation--a neglected entity? Crit Care 2009 13 215 10.1186/cc7794 19638180 PMC2717412

[B11] Bloemsma GC Dokter J Boxma H Oen IM Mortality and causes of death in a burn centre Burns 2008 34 1103 1107 10.1016/j.burns.2008.02.010 18538932

[B12] Chipp E Milner CS Blackburn AV Sepsis in burns: a review of current practice and future therapies Ann Plast Surg 2010 65 228 236 10.1097/SAP.0b013e3181c9c35c 20606586

[B13] Gatson JW Maass DL Simpkins JW Idris AH Minei JP Wigginton JG Estrogen treatment following severe burn injury reduces brain inflammation and apoptotic signaling J Neuroinflammation 2009 6 30 10.1186/1742-2094-6-30 19849845 PMC2774304

[B14] Reyes R Jr Wu Y Lai Q Mrizek M Berger J Jimenez DF Barone CM Ding Y Early inflammatory response in rat brain after peripheral thermal injury Neurosci Lett 2006 407 11 15 10.1016/j.neulet.2006.07.071 16935421

[B15] Amantea D Nappi G Bernardi G Bagetta G Corasaniti MT Post-ischemic brain damage: pathophysiology and role of inflammatory mediators Febs J 2009 276 13 26 10.1111/j.1742-4658.2008.06766.x 19087196

[B16] Barbizan R Oliveira AL Impact of acute inflammation on spinal motoneuron synaptic plasticity following ventral root avulsion J Neuroinflammation 2010 7 29 10.1186/1742-2094-7-29 20441580 PMC2874529

[B17] Djukic M Mildner A Schmidt H Czesnik D Bruck W Priller J Nau R Prinz M Circulating monocytes engraft in the brain, differentiate into microglia and contribute to the pathology following meningitis in mice Brain 2006 129 2394 2403 10.1093/brain/awl206 16891321

[B18] D'Mello C Le T Swain MG Cerebral microglia recruit monocytes into the brain in response to tumor necrosis factoralpha signaling during peripheral organ inflammation J Neurosci 2009 29 2089 2102 10.1523/JNEUROSCI.3567-08.2009 19228962 PMC6666330

[B19] Vendrame M Gemma C de Mesquita D Collier L Bickford PC Sanberg CD Sanberg PR Pennypacker KR Willing AE Anti-inflammatory effects of human cord blood cells in a rat model of stroke Stem Cells Dev 2005 14 595 604 10.1089/scd.2005.14.595 16305344

[B20] Yin HL Stossel TP Control of cytoplasmic actin gel-sol transformation by gelsolin, a calcium-dependent regulatory protein Nature 1979 281 583 586 10.1038/281583a0 492320

[B21] Janmey PA Lind SE Capacity of human serum to depolymerize actin filaments Blood 1987 70 524 530 3038216

[B22] Goncalves AF Dias NG Moransard M Correia R Pereira JA Witke W Suter U Relvas JB Gelsolin is required for macrophage recruitment during remyelination of the peripheral nervous system Glia 2010 58 706 715 20014276 10.1002/glia.20956

[B23] Morley SC Sung J Sun GP Martelli MP Bunnell SC Bierer BE Gelsolin overexpression alters actin dynamics and tyrosine phosphorylation of lipid raft-associated proteins in Jurkat T cells Mol Immunol 2007 44 2469 2480 10.1016/j.molimm.2006.09.024 17178161 PMC1945820

[B24] Jordan JR Moore EE Damle SS Eckels P Johnson JL Roach JP Redzic JS Hansen KC Banerjee A Gelsolin is depleted in post-shock mesenteric lymph J Surg Res 2007 143 130 135 10.1016/j.jss.2007.04.017 17950082 PMC2695490

[B25] Osborn TM Verdrengh M Stossel TP Tarkowski A Bokarewa M Decreased levels of the gelsolin plasma isoform in patients with rheumatoid arthritis Arthritis Res Ther 2008 10 R117 10.1186/ar2520 18822171 PMC2592804

[B26] Lee PS Patel SR Christiani DC Bajwa E Stossel TP Waxman AB Plasma gelsolin depletion and circulating actin in sepsis: a pilot study PLoS One 2008 3 e3712 10.1371/journal.pone.0003712 19002257 PMC2577888

[B27] Wang H Cheng B Chen Q Wu S Lv C Xie G Jin Y Fang X Time course of plasma gelsolin concentrations during severe sepsis in critically ill surgical patients Crit Care 2008 12 R106 10.1186/cc6988 18706105 PMC2575595

[B28] Lee PS Waxman AB Cotich KL Chung SW Perrella MA Stossel TP Plasma gelsolin is a marker and therapeutic agent in animal sepsis Crit Care Med 2007 35 849 855 10.1097/01.CCM.0000253815.26311.24 17205019

[B29] Christofidou-Solomidou M Scherpereel A Solomides CC Christie JD Stossel TP Goelz S DiNubile MJ Recombinant plasma gelsolin diminishes the acute inflammatory response to hyperoxia in mice J Investig Med 2002 50 54 60 10.2310/6650.2002.33518 11813829

[B30] Carlson DL Maass DL White J Sikes P Horton JW Caspase inhibition reduces cardiac myocyte dyshomeostasis and improves cardiac contractile function after major burn injury J Appl Physiol 2007 103 323 330 10.1152/japplphysiol.01255.2006 17431085

[B31] Rothenbach PASJ Dahl B O'Keefe E Yamamoto Y Lee WM Horton JWYH Turnage RH Recombinant plasma gelsolin infusion attenuates burn-induced pulmonary microvascular dysfunction J Appl Physiol 2004 96 23 31 10.1152/japplphysiol.01074.2002 12730154

[B32] Livak KJ Schmittgen TD Analysis of relative gene expression data using real-time quantitative PCR and the 2(-Delta Delta C(T)) Method Methods 2001 25 402 408 10.1006/meth.2001.1262 11846609

[B33] Wang H Bloom O Zhang M Vishnubhakat JM Ombrellino M Che J Frazier A Yang H Ivanova S Borovikova L HMG-1 as a late mediator of endotoxin lethality in mice Science 1999 285 248 251 10.1126/science.285.5425.248 10398600

[B34] Abraham E Arcaroli J Carmody A Wang H Tracey KJ HMG-1 as a mediator of acute lung inflammation J Immunol 2000 165 2950 2954 10975801 10.4049/jimmunol.165.6.2950

[B35] Bonaldi T Talamo F Scaffidi P Ferrera D Porto A Bachi A Rubartelli A Agresti A Bianchi ME Monocytic cells hyperacetylate chromatin protein HMGB1 to redirect it towards secretion Embo J 2003 22 5551 5560 10.1093/emboj/cdg516 14532127 PMC213771

[B36] Yang QW Lu FL Zhou Y Wang L Zhong Q Lin S Xiang J Li JC Fang CQ Wang JZ HMBG1 mediates ischemia-reperfusion injury by TRIF-adaptor independent Toll-like receptor 4 signaling J Cereb Blood Flow Metab 2010 31 593 605 20700129 10.1038/jcbfm.2010.129 PMC3049514

[B37] Scaffidi P Misteli T Bianchi ME Release of chromatin protein HMGB1 by necrotic cells triggers inflammation Nature 2002 418 191 195 10.1038/nature00858 12110890

[B38] Streit WJ Conde JR Fendrick SE Flanary BE Mariani CL Role of microglia in the central nervous system's immune response Neurol Res 2005 27 685 691 16197805 10.1179/016164105X49463a

[B39] Hara H Kataoka S Anan M Ueda A Mutoh T Tabira T The therapeutic effects of the herbal medicine, Juzen-taiho-to, on amyloid-beta burden in a mouse model of Alzheimer's disease J Alzheimers Dis 2010 20 427 439 20164567 10.3233/JAD-2010-1381

[B40] Shi FD Piao WH Kuo YP Campagnolo DI Vollmer TL Lukas RJ Nicotinic attenuation of central nervous system inflammation and autoimmunity J Immunol 2009 182 1730 1739 19155522 10.4049/jimmunol.182.3.1730

[B41] Hirko AC Meyer EM King MA Hughes JA Peripheral transgene expression of plasma gelsolin reduces amyloid in transgenic mouse models of Alzheimer's disease Mol Ther 2007 15 1623 1629 10.1038/sj.mt.6300253 17609655

[B42] Dickstein DL Biron KE Ujiie M Pfeifer CG Jeffries AR Jefferies WA Abeta peptide immunization restores blood-brain barrier integrity in Alzheimer disease Faseb J 2006 20 426 433 10.1096/fj.05-3956com 16507760

[B43] Messaris E Memos N Chatzigianni E Konstadoulakis MM Menenakos E Katsaragakis S Voumvourakis C Androulakis G Time-dependent mitochondrial-mediated programmed neuronal cell death prolongs survival in sepsis Crit Care Med 2004 32 1764 1770 10.1097/01.CCM.0000135744.30137.B4 15286556

[B44] Sharshar T Gray F Lorin de la Grandmaison G Hopkinson NS Ross E Dorandeu A Orlikowski D Raphael JC Gajdos P Annane D Apoptosis of neurons in cardiovascular autonomic centres triggered by inducible nitric oxide synthase after death from septic shock Lancet 2003 362 1799 1805 10.1016/S0140-6736(03)14899-4 14654318

[B45] Spera PA Ellison JA Feuerstein GZ Barone FC IL-10 reduces rat brain injury following focal stroke Neurosci Lett 1998 251 189 192 10.1016/S0304-3940(98)00537-0 9726375

[B46] van Gool WA van de Beek D Eikelenboom P Systemic infection and delirium: when cytokines and acetylcholine collide Lancet 2010 375 773 775 10.1016/S0140-6736(09)61158-2 20189029

[B47] Depino AM Earl C Kaczmarczyk E Ferrari C Besedovsky H del Rey A Pitossi FJ Oertel WH Microglial activation with atypical proinflammatory cytokine expression in a rat model of Parkinson's disease Eur J Neurosci 2003 18 2731 2742 10.1111/j.1460-9568.2003.03014.x 14656322

[B48] Zudaire E Martinez A Cuttitta F Adrenomedullin and cancer Regul Pept 2003 112 175 183 10.1016/S0167-0115(03)00037-5 12667640

[B49] Duan H Chai J Sheng Z Yao Y Yin H Liang L Shen C Lin J Effect of burn injury on apoptosis and expression of apoptosis-related genes/proteins in skeletal muscles of rats Apoptosis 2009 14 52 65 10.1007/s10495-008-0277-7 19009350

[B50] Zhang JP Ying X Liang WY Luo ZH Yang ZC Huang YS Wang WC Apoptosis in cardiac myocytes during the early stage after severe burn J Trauma 2008 65 401 408 discussion 408 10.1097/TA.0b013e31817cf732 18695479

[B51] Geissmann F Manz MG Jung S Sieweke MH Merad M Ley K Development of monocytes, macrophages, and dendritic cells Science 2010 327 656 661 10.1126/science.1178331 20133564 PMC2887389

[B52] Nahrendorf M Pittet MJ Swirski FK Monocytes: protagonists of infarct inflammation and repair after myocardial infarction Circulation 121 2437 2445 10.1161/CIRCULATIONAHA.109.916346 PMC2892474 20530020

[B53] Roy A Hooper DC Lethal silver-haired bat rabies virus infection can be prevented by opening the blood-brain barrier J Virol 2007 81 7993 7998 10.1128/JVI.00710-07 17507463 PMC1951307

[B54] Witke W Sharpe AH Hartwig JH Azuma T Stossel TP Kwiatkowski DJ Hemostatic, inflammatory, and fibroblast responses are blunted in mice lacking gelsolin Cell 1995 81 41 51 10.1016/0092-8674(95)90369-0 7720072

[B55] Lin ECJ Leak RK Perez RG Zigmond MJ Rapid activation of ERK by 6-hydroxydopamine promotes survival of dopaminergic cells J Neurosci Res 2008 86 108 117 10.1002/jnr.21478 17847117

[B56] Liu B Zhang H Xu C Yang G Tao J Huang J Wu J Duan X Cao Y Dong J Neuroprotective effects of icariin on corticosterone-induced apoptosis in primary cultured rat hippocampal neurons Brain Res 2010 1375 59 67 21182828 10.1016/j.brainres.2010.12.053

[B57] Wakade C Khan MM De Sevilla LM Zhang QG Mahesh VB Brann DW Tamoxifen neuroprotection in cerebral ischemia involves attenuation of kinase activation and superoxide production and potentiation of mitochondrial superoxide dismutase Endocrinology 2008 149 367 379 17901229 10.1210/en.2007-0899 PMC2194601

[B58] Zhang JZ Jing L Ma Y Guo FY Chang Y Li PA Monosialotetrahexosy-1 ganglioside attenuates diabetes-enhanced brain damage after transient forebrain ischemia and suppresses phosphorylation of ERK1/2 in the rat brain Brain Res 2010 1344 200 208 20546707 10.1016/j.brainres.2010.05.044 PMC2900456

[B59] Alessandrini A Namura S Moskowitz MA Bonventre JV MEK1 protein kinase inhibition protects against damage resulting from focal cerebral ischemia Proc Natl Acad Sci USA 1999 96 12866 12869 10.1073/pnas.96.22.12866 10536014 PMC23136

[B60] Nguyen DN Spapen H Su F Schiettecatte J Shi L Hachimi-Idrissi S Huyghens L Elevated serum levels of S-100 beta protein and neuron-specific enolase are associated with brain injury in patients with severe sepsis and septic shock Crit Care Med 2006 34 1967 1974 10.1097/01.CCM.0000217218.51381.49 16607230

[B61] Patenaude J D'Elia M Hamelin C Garrel D Bernier J Burn injury induces a change in T cell homeostasis affecting preferentially CD4+ T cells J Leukoc Biol 2005 77 141 150 15542542 10.1189/jlb.0703314

[B62] Smith DBJP Herbert TJ Lind SE Quantitative measurement of plasma gelsolin and its incorporation into fibrin clots J Lab Clin Med 1987 110 189 195 3036979

